# A Manual of Dietetics

**Published:** 1886-10

**Authors:** 


**A Manual of Dietetics.** By J. MILNER FOTHERGILL, M. D., EDIN., Physician to the City of London Hospital for Diseases of the Chest (Victoria Park). Hon. M. D. Rush Medical College, Chicago, Ill., Foreign Associate Fellow of the College of Physicians, Philadelphia. 8vo. extra muslin. 255 pages. Price, $2.50. New York: William Wood & Co.

This is a new and original work. Its distinguished author has devoted much time to the study of dietetics, and in view of the modern advances in our knowledge of the physiology of digestion, its appearance is most timely. Proper feeding in disease is often more important than the administration of drugs, and this book admirably covers a most important, and up to the present, neglected field. The subject is treated in a masterly way, and the style is so sprightly that few physicans taking it up will not be interested as well as instructed. We must heartily advise our readers to buy the book.

